# Ki67 in Breast Cancer Assay: An Ad Hoc Testing Recommendation from the Canadian Association of Pathologists Task Force

**DOI:** 10.3390/curroncol30030233

**Published:** 2023-03-06

**Authors:** Hala Faragalla, Anna Plotkin, Penny Barnes, Fang-I Lu, Zuzana Kos, Anna Marie Mulligan, Anita Bane, Sharon Nofech Mozes

**Affiliations:** 1 Department of Laboratory Medicine, St. Michael’s Hospital, Toronto, ON M5B 1W8, Canada; 2 Department of Laboratory Medicine and Molecular Diagnostics Sunnybrook Health Sciences Center, Toronto, ON M4N 3M5, Canada; 3 Department of Pathology and Laboratory Medicine, Nova Scotia Health Authority, Halifax, NS B3H 2E2, Canada; 4 Department of Pathology, BC Cancer, Vancouver, BC V5Z 4E6, Canada; 5 Department of Laboratory Medicine, University Health Network, Toronto, ON M5T 2S8, Canada

**Keywords:** Ki67 breast cancer, MonarchE trial, advanced early-stage breast cancer

## Abstract

Ki67, a marker of cellular proliferation, is commonly assessed in surgical pathology laboratories. In breast cancer, Ki67 is an established prognostic factor with higher levels associated with worse long-term survival. However, Ki67 IHC is considered of limited clinical use in breast cancer management largely due to issues related to standardization and reproducibility of scoring across laboratories. Recently, both the American Food and Drug Administration (FDA) and Health Canada have approved the use of abemaciclib (CDK4/6 inhibitor) for patients with HR+/HER2: high-risk early breast cancers in the adjuvant setting. Health Canada and the FDA have included a Ki67 proliferation index of ≥20% in the drug monograph. The approval was based on the results from monarchE, a phase III clinical trial in early-stage chemotherapy-naïve, HR+, HER2 negative patients at high risk of early recurrence. The study has shown significant improvement in invasive disease-free survival (IDFS) with abemaciclib when combined with adjuvant endocrine therapy at two years. Therefore, there is an urgent need by the breast pathology and medical oncology community in Canada to establish national guideline recommendations for Ki67 testing as a predictive marker in the context of abemaciclib therapy consideration. The following recommendations are based on previous IKWG publications, available guidance from the monarchE trial and expert opinions. The current recommendations are by no means final or comprehensive, and their goal is to focus on its role in the selection of patients for abemaciclib therapy. The aim of this document is to guide Canadian pathologists on how to test and report Ki67 in invasive breast cancer. Testing should be performed upon a medical oncologist’s request only. Testing must be performed on treatment-naïve tumor tissue. Testing on the core biopsy is preferred; however, a well-fixed resection specimen is an acceptable alternative. Adhering to ASCO/CAP fixation guidelines for breast biomarkers is advised. Readout training is strongly recommended. Visual counting methods, other than eyeballing, should be used, with global rather than hot spot assessment preferred. Counting 100 cells in at least four areas of the tumor is recommended. The Ki67 scoring app developed to assist pathologists with scoring Ki67 proposed by the IKWG, available for free download, may be used. Automated image analysis is very promising, and laboratories with such technology are encouraged to use it as an adjunct to visual counting. A score of <5 or >30 is more robust. The task force recommends that the results are best expressed as a continuous variable. The appropriate antibody clone and staining protocols to be used may take time to address. For the time being, the task force recommends having tonsils/+pancreas on-slide control and enrollment in at least one national/international EQA program. Analytical validation remains a pending goal. Until the data become available, using local ki67 protocols is acceptable. The task force recommends participation in upcoming calibration and technical validation initiatives.

## 1. Background

Ki67, a marker of cellular proliferation, is commonly assessed in surgical pathology laboratories using immunohistochemistry (IHC). In breast cancer, Ki67 is an established prognostic factor with higher levels associated with worse long-term survival [1,2,3]. In the neoadjuvant setting, a higher Ki67 proliferation index is associated with greater response to neoadjuvant chemotherapy and worse long-term survival [4]. Moreover, “on treatment” Ki67 assessment in postmenopausal women with hormone-sensitive breast cancer who received neoadjuvant endocrine therapy can provide information on 5-year survival and possibly identify patients who are more likely to benefit from the addition of chemotherapy [5]. Nonetheless, its clinical utility in this setting is yet to be established. Many of the commercially available multigene prognostic signatures used to estimate an individual patient’s risk of breast cancer recurrence measure proliferation as part of the assay [6]. However, Ki67 IHC is considered of limited clinical use in breast cancer management largely due to issues related to standardization and reproducibility of scoring across laboratories [7,8,9].

Approximately 70–75% of breast cancer is hormone receptor (HR) positive and HER2 negative. Endocrine therapies such as selective estrogen receptor modulators (SERM), aromatase inhibitors (AI) and selective estrogen receptor degraders (SERD) are considered the backbone of systemic therapy with the addition of chemotherapy in patients at significant risk of distant recurrence. Recent randomized clinical trials (TAILORx and RxPONDER) have shown that not all patients with this type of breast cancer benefit from chemotherapy. Another class of systemic therapy in this arena includes the cyclin-dependent kinases 4/6 (CDK4/6) inhibitors (palbociclib, ribociclib, and abemaciclib) that regulate the cellular transition from the G1 phase of the cell cycle to the S phase, blocking proliferation of cancer cells by inducing cellular arrest in G1. In the metastatic setting, they have been shown to provide longer progression-free survival than estrogen modulation therapy alone [10].

Recently, both the American Food and Drug Administration (FDA) and Health Canada have approved the use of abemaciclib (CDK4/6 inhibitor) for patients with HR+/HER2- high-risk early breast cancers in the adjuvant setting. The approval was based on the results from monarchE, a phase III clinical trial in early-stage chemotherapy-naïve, HR+, HER2-, node-positive breast cancer patients at high risk of early recurrence. The study has shown significant improvement in invasive disease-free survival (IDFS) with abemaciclib when combined with adjuvant endocrine therapy at two years, with a larger absolute benefit in Ki67 high tumors [11] while the results reporting overall survival (OS) at 42 months did not show a significant difference between the two arms [12](abemaciclib plus endocrine therapy versus endocrine therapy alone). Although the monarchE data showed that Ki67 is prognostic for the outcome, it had a limited role as a predictive marker of response to abemaciclib [11]. Nevertheless, Health Canada and the FDA have included a Ki67 proliferation index of ≥20% in the drug monograph. Notably, a Ki67 score was not included in abemaciclib registration in the European Union, the UK, or Japan. A recent study analyzed data from cohort 1 patients in the monarchE study and showed the benefits of abemaciclib plus endocrine therapy in HR+/HER2-high-risk early breast cancer patients based on clinicopathological features only [13].

Consequently, ASCO recommended that despite the limitations associated with Ki67 testing, a patient with node-positive breast cancer at high risk of recurrence and a Ki67 score of ≥20%, as determined by an FDA-approved test, may be offered two years of abemaciclib plus endocrine therapy (recommendations 1.2) [14].

This document is intended to guide Canadian pathologists on how to test and report Ki67 in invasive breast cancer. The discussion of whether the Ki67 score should be used as a criterion for selecting patients for abemaciclib is beyond its scope.

Health Canada’s approval of abemaciclib with Ki67 ≥20% as determined by a companion diagnostic has led to an immediate action by the Canadian Association of Pathologists breast interest group to provide national recommendations to the pathology community regarding testing and reporting on Ki67 in breast cancer, notwithstanding the known limitations.

The International Ki67 in Breast Cancer Working Group (IKWG) identified multiple factors, including preanalytical (collection, processing and archiving), analytical (staining and analysis) and post-analytical (readout/scoring), that caused variability in Ki67 scoring [2,15,16,17,18]. Over the past decade, the group has taken a stepwise approach to address the technical and scoring aspects of the Ki67 assessment, aiming to enhance interlaboratory and interobserver reproducibility.

The work from the IKWG showed that reproducibility improved when the criteria for readout/scoring were uniformly applied by trained, qualified readers and highlighted the potential benefit of automated scoring using imaging analysis tools [16]. Despite this, a kappa value of 0.6 (moderate agreement) has been demonstrated for interobserver agreement in the 10–20% Ki67 range in multiple studies [16,19], and substantial interobserver variability has been observed in intermediate-grade carcinomas with a Ki67 value between 5–30%, with kappa values of 0.04–0.14 reported [20].

In the monarchE study, the Ki67 index was assessed centrally with the Ki67 PharmDx (Agilent, ready-to-use) assay on the Omnis platform. The method of scoring in terms of the threshold for positivity and the number of cells counted varies slightly from IKWG recommendations [21]. The Agilent Ki67 PharmDx assay performed on the Omnis platform is not available in Canada, and thus one key issue to be resolved is whether the FDA-approved assay is comparable with other laboratory-developed assays used in Canadian laboratories. Specifically, it is unclear at this time whether the 20% cutoff applied in the monarchE trial would be applicable when using other antibodies, kits and platforms. While the answers to these extremely important analytical questions are still pending, Canadian pathologists are being asked to assess Ki67 by the medical oncology community in order to access this potentially useful therapy.

Therefore, there is an urgent need by the breast pathology and medical oncology community in Canada to establish national guideline recommendations for Ki67 testing as a predictive marker in the context of abemaciclib therapy consideration.

The following recommendations are based on previous IKWG publications, available guidance from the monarchE trial and expert opinions. The current recommendations are by no means final or comprehensive, and their goal is to focus on its role in the selection of patients for abemaciclib therapy.

## 2. Questions to Address

When to test: Should Ki67 testing be performed by individual request or as a reflex test in invasive breast cancer? At the present time, it is recommended that Ki67 be performed at the clinician’s request in patients being considered for treatment. There is no need or capacity for reflex testing. At present, based on ASCO/CAP guidelines, the only clinical utility of Ki67 testing in breast carcinoma is when systemic therapy with abemaciclib is considered in patients with clinically HR+/HER2-high-risk early breast cancers. According to statistics Canada (https://www150.statcan.gc.ca/n1/pub/82-003-x/2018012/article/00003/c-g/c-g03-eng.htm, accessed on 15 February 2023), the majority of newly diagnosed breast cancer patients are diagnosed at an early stage and in these patients Ki67 testing has no clinical role.

With a growing workload and limited resources, there is no justification for implementing universal upfront testing.

Which tissue to test: Should testing be performed (i) in primary or metastatic carcinoma, (ii) in treatment-naïve or post-treatment tumor, (iii) on the initial core biopsy or resection specimen? Testing should be performed on treatment-naïve breast cancer in breast tissue. If a patient received neoadjuvant systemic therapy, the initial core biopsy must be used. For patients initially treated by surgery, the task force recommends testing a core biopsy when possible, but a whole well-fixed section from untreated breast tissue is an acceptable alternative. There is a high but not perfect concordance between paired core biopsies and whole sections from resection specimens, with the scores being systematically higher in core biopsies, possibly due to more controlled preanalytical variables [22,23,24]. Tissue fixed in alcohol-based fixatives, often used in cytology preparation, as well as decalcified tissue, should be avoided.

Preanalytical variables: Preanalytical variables affect Ki67. These include the type of tissue (biopsy versus resection), cold ischemic time, fixation time [25] and long-term storage. Notably, the Ki67 index decreases significantly with prolonged fixation compared to other breast cancer markers [25]. The recommendation is to follow ASCO/CAP guidelines for HR/HER2, which include adhering to cold ischemic times of less than 1 h and a fixation duration of between 6–72 h with neutral buffered formalin (NBF) as a fixative [26].

Centralized testing vs. widespread testing: A laboratory can locally test if there are at least two designated qualified readers with the appropriate caseload, appropriate training and growing experience. Laboratories with a very low case load should discuss and consider referral.

Caseload per reader: Currently, there is no suggested minimum caseload per reader per year in the available literature. To maintain competency, laboratories with a very low caseload may consider appointing a designated reader or referral to a central laboratory. Pathologists with very few requests for Ki67 scoring in breast cancer may enrich their exposure through readout modules offered by CBQA (https://cbqareadout.ca/; Breast Ki67 module is under construction) (15 February 2023) and other proficiency testing modules such as “The Canadian Pathology Quality Assurance—Assurance qualité canadienne en pathologie (CPQA-AQCP)”, a non-profit corporation dedicated to ensuring quality biomarker testing in medical diagnostic laboratories. Pathologists that are uncomfortable with scoring Ki67 in breast cancer are encouraged to send it to another lab with appropriate expertise.

Readout Training: Online training provided by the IKWG (http://www.gpec.ubc.ca:8080/tmadb-0.1/calibrator/index, last accessed 1 March 2023), or Canadian Biomarkers Quality Assurance site (https://cap-acp.org/Proficiency-Testing-Biomarker-Readout.php, last accessed 1 March 2023) is recommended, once it becomes available.

The IKWG has stressed scoring differences as an important cause of variability across laboratories and observers. They showed that in the absence of a standardized staining and scoring methodology, interlaboratory reproducibility was moderate (central staining intraclass correlation coefficient (ICC) = 0.71, local staining ICC = 0.59) [7]. In their phase 2 study, an online calibration exercise (for color thresholds and tumor cell selection) and standardized nuclei counting methods were implemented. When the same section was scored, the ICC was 0.94, and when a different section was scored, the ICC was 0.92 [8].

Analytical validation: In the absence of a clinical validation set, analytical validation is acceptable and remains a pending goal. The task force recommends participation in upcoming calibration and technical validation initiatives.

The antibody clone and staining protocols to be used are areas in which gaps exist, and these may take time to address. For the time being, the task force recommends having tonsils/+pancreas on-slide control and enrollment in at least one national or international EQA program. A recent publication describes a cell line microarray system in which mixes of human Karpas 299 or Jurkat cells (Ki67+) with Sf9 (Spodoptera frugiperda) (Ki67−) cells are present in incremental standardized ratios [27]. This cell line standardization system may aid in normalizing staining variability between different antibodies.

Which antibody clone to use: Ki67 was evaluated in the monarchE study using the MIB-1 clone by Agilent. This comes in a ready-to-use kit performed on the Dako Omnis platform using a validated automated staining protocol. Some important questions to be asked are whether the kit used in the clinical trial performance is comparable to other available Ki67 assays and how the 20% threshold would be applicable to other assays and kits. A UK study examined data from 374 laboratories as part of an external quality assessment. Their study revealed that the 30–39 (Ventana) and K2 Leica Biosystems clones were associated with the highest mean scores. They also showed that MIB-1 was more likely to be associated with good quality staining results when it is used with the Agilent Dako antigen retrieval systems, detection, and staining platform. Their data demonstrated not only the importance of the antibody clone used but also other methodological factors [28]. The IKWG and a UK study have acknowledged MIB-1 as a widely clinically validated Ki67 antibody [17,28]. Until Ki67 PharmDx (Agilent, ready to use) assay on the Omnis platform and calibration and technical validation studies are available, the Task Force recommends following local ki67 antibody and staining protocols.

**Figure 1:**
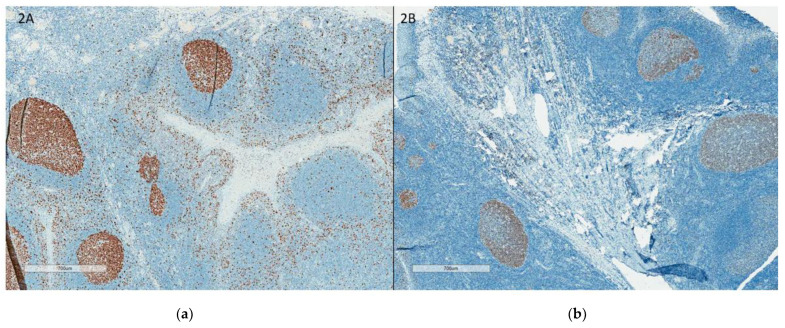
(**a**) Tonsil with good staining, accepted external control; (**b**): Tonsil with weak staining, rejected external control. (**a**,**b**) illustrates the tonsil as external control with good staining (**a**) and with weak staining (**b**).

**Figure 2:**
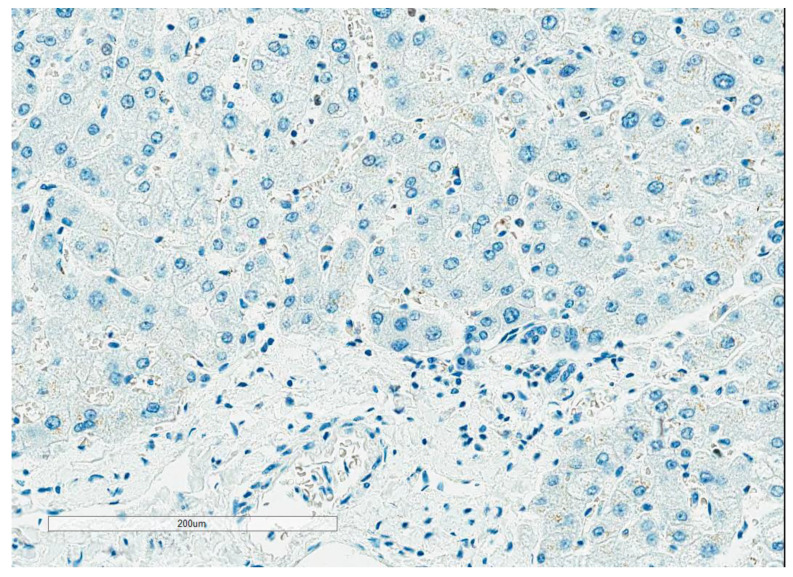
Liver as Ki67 external negative control. Figure 2 illustrates the liver as Ki67 external negative control.

Staining protocols: One of the main challenges in Ki67 assessment is the lack of negative control, as all tissues have some proliferation that is highlighted by Ki67 IHC. This has resulted in different dilutions of antibodies leading to different intensities of nuclear staining across different laboratories. NordiQC recommends using the tonsil as an external positive and negative control. They recommend strong to moderate staining of all B cells in the dark zones, with weak to moderate staining of B cells in the light zones and no staining of the mantle zone (Figure 1). With the liver and pancreas as supplementary negative controls, <1% of hepatocytes and cells of the exocrine pancreas should be positive (Figure 2).

Method of assessment: Visual methods and/or automated image analysis may be used.

Visual methods:

Eyeballing: this has been shown to be inaccurate and poorly reproducible [29,30].

Global assessment (per IKWG): counting four sets of 100 cells, attempting to derive an average score across the tumor [16].

Weighted global assessment (per IKWG): as for global assessment but weighted according to the estimated percentage of the total tumor area covered by each of high, medium, low, or negligible Ki67 staining levels (of note, this method gave the best concordance between observers [18]).

Hot spot assessment: counting a single field of 500 cells in the tumor area with the highest rate.

The IKWG, in their Phase Three validation study, compared three Ki67 scoring methods (global, weighted global and hot spot) on 30 core biopsies. Only the unweighted global scoring method met the prespecified criterion for success, whereas the weighted global and the hot spot methods failed to meet this criterion marginally [18]. In phase 3b, the IKWG whole tissue sections were stained, and two scoring methods were compared: global and hot spots. They showed similar results with the global method meeting the prespecified criterion for success, while the hot spot assessment did not. A high intraclass correlation coefficient between two scorers in both global weighted (ICC 0.915, 95% confidence interval) and global unweighted (ICC 0.920, 95% confidence interval) assessment has been demonstrated [31]. A free app (Ki67 scoring app) developed by the IKWG is available to assist with Ki67 weighted and unweighted global assessment (https://play.google.com/store/apps/details?id=ca.ubc.gpec.ki67counter&hl=en_CA&gl=US) (accessed on 15 February 2023).

In the monarchE study, the entire tissue was scored, and if hot spots were present in the tissue section, they were included in the final Ki67 score [21].

**Figure 3:**
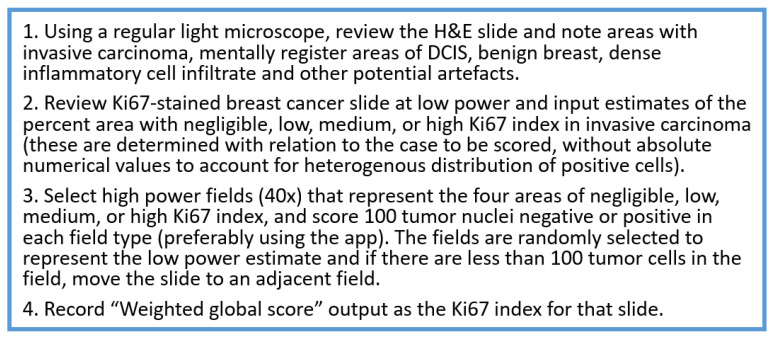
How to select 4 high power fields for global Ki67 assessment across the tumor.

The task force recommends using IKWG’s global assessment method to reflect an average score across the tumor. Figure 3 illustrates how to select four high-power fields for global Ki67 assessment across the tumor. If the case is close to the 20% cutoff, reporting both weighted and unweighted (%) scores is recommended. Counting additional cells may be useful in borderline cases (see below).

Image analysis systems: Laboratories with access to such technology are encouraged to use it in association with global assessment methods. The open-source QuPath platform demonstrated an excellent interclass correlation that outperformed visual counting [32].

The IKWG performed a Ki67 image analysis study which included 30 breast carcinomas, 7 different scanners and 10 platforms. Automated assessments of average and maximum counts were calculated. The intraclass correlation coefficient (ICC) for average scores was 0.83 (for 16 operators), and the ICC for maximum scores was 0.63 (for 10 operators) [33].

Another Ki67 reproducibility study was performed on three platforms: QuPath, HALO, and Quant Center. They demonstrated excellent reproducibility and a high intraclass correlation coefficient (of an average of >0.9) between and within platforms, suggesting that image analysis could be easily standardized to provide highly reproducible Ki67 scores [32].

What constitutes a positive nucleus: One of the most common challenges in scoring is what constitutes positive nuclear staining. The FDA-approved criteria used in the monarchE trial consider a nucleus to be positively stained if it meets the following criteria: (1) The signal must be unequivocally brown; (2) The staining must correspond to a nucleus; (3) The staining must cover the whole distribution within the nucleus; (4) The staining must correspond to viable, nonapoptotic cells [21]. In situ carcinoma and areas of necrosis are not to be scored, and grey nuclei are excluded [21]. It should be noted that the IKWG defined positive nuclei as any brown color above the background [17]. The practical significance of distinguishing between what constitutes a light brown (1+) positive tumor nucleus (lower staining threshold) versus grey (zero) is yet to be established.

**Figure 4:**
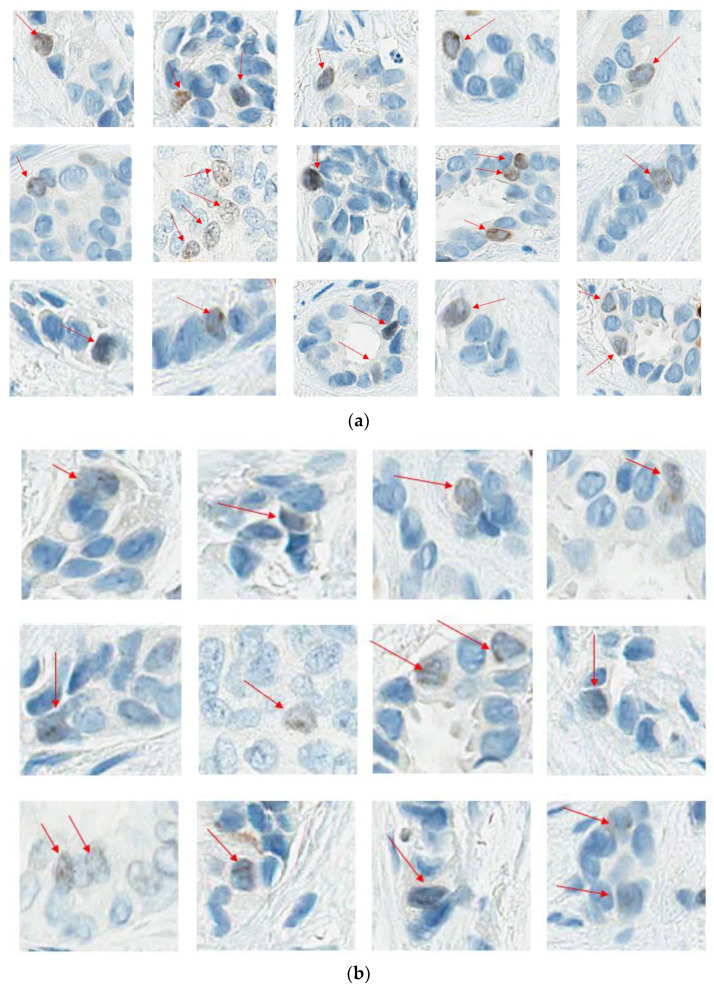

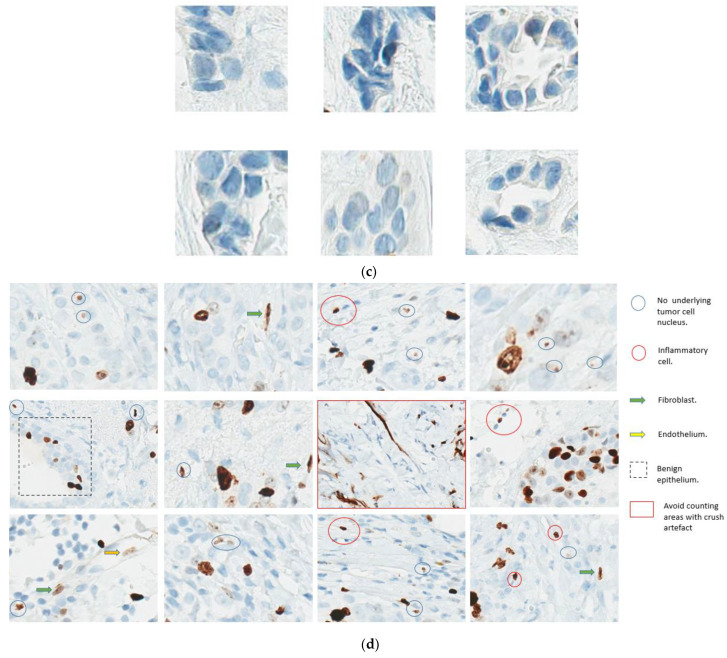
(**a**). Ki67 Positive nuclei; (**b**): Ki67 Light brown positive nuclei; (**c**): Ki67 Negative nuclei. (**d**): Don’t count these. (**a,b**) illustrate the spectrum of Ki67-positive nuclei. As per IKWG, any staining above the background is considered positive. (**c**) Illustrates Ki67 negative nuclei. (**d**). Illustrates what not to count when counting Ki67.

At this point, the task force recommends following the IKWG definition, i.e., any nuclear staining above background, as it is proven to be reproducible across multiple readers. Figure 4a–c illustrate examples of positive and negative nuclei and Figure 4d examples of signals that should not be counted.

How many cells should be counted: The task force recommends counting 400 tumor nuclei.

The monarchE trial recommends that a sample should contain a minimum of 200 viable tumor cells to be considered suitable for analysis. The number of tumor cells counted is not specified, but they report scoring all viable tumor cells in a tissue section. The IKWG recommends counting four fields of 100 tumor cells to reflect heterogeneity in nuclear staining in a particular tumor. Visual counting under a light microscope or from a digital image is recommended. The percentage of positively stained nuclei over the total number of invasive cells counted should be recorded.

A Ki67 proliferation index of <5 or >30 is more robust. In cases within the 5–30% range, consider counting another set of 400 cells, show it to a second rater, or, if available, perform an automated assessment using an image analysis system. If one count is ≥20%, this should be reported to facilitate access to the drug. Avoid counting cells that are not invasive cancer (normal epithelium, DCIS or stromal/immune cells). Areas with necrosis, edge effects and fixation and processing artifact should not be scored.

How to express the results: Ki67 may be reported as a continuous variable or as ≥20% or <20, based on the proposed cutoff for treatment eligibility. The task force recommends reporting as a continuous variable rather than categorical, as Ki67 is likely to have a continuous biologic effect [16].

## 3. Summary and Recommendations

This is the first Canadian guideline recommendation for pathologists for Ki67 scoring in breast cancer.

- It is recommended that testing upon medical oncologist request only is indicated.
- Testing must be performed on treatment-naïve tumor tissue. Testing on the core biopsy is preferred; however, a well-fixed resection specimen is an acceptable alternative.
- Adhering to ASCO/CAP fixation guidelines for breast biomarkers is advised, including cold ischemic times of <1 h, duration of fixation of 6 to 72 h and using neutral buffered formalin only as a fixative.
- Readout training is strongly recommended. Visual counting methods, other than eyeballing, should be used, with global rather than hot spot assessment preferred. Counting 100 cells in at least four areas of the tumor is recommended. It is suggested to evaluate Ki67 at 40× magnification to capture weakly staining nuclei. The Ki67scoring app developed to assist pathologists with scoring Ki67 using the standardized scoring method proposed by the IKWG, available for free download, may be used (https://play.google.com/store/apps/details?id=ca.ubc.gpec.ki67counter&hl=en_CA&gl=US, accessed on 15 February 2023). Automated image analysis is very promising and can be easily standardized, and laboratories with such technology are encouraged to use it as an adjunct to visual counting.
- A score of <5 or >30 is more robust. In cases within the 5–30% range, consider counting another set of 400 cells, have a second reader count, or refer to an image analysis system.
- The task force recommends that the results are best expressed as a continuous variable and reported in a synoptic format using the CAP checklist or the suggested format (Appendix A).
- The appropriate antibody clone and staining protocols to be used may take time to address. For the time being, the task force recommends having tonsils/+pancreas on-slide control and enrollment in at least one national/international EQA program.
- Analytical validation remains a pending goal. Until the data become available, using local Ki67 protocols is acceptable. The task force recommends participation in upcoming calibration and technical validation initiatives. When reporting Ki67, consider adding a disclaimer indicating that the assay is currently not validated for prognostic/predictive purposes in breast cancer, and calibration/validation will be pursued once they become available.

## Data Availability

Not applicable.

## Appendix A. Synoptic Report Template

Specimen

    Specimen#

Specimen Type#

Test Performed: Ki67

    Block #

    Tumor Type:

Results: Ki67

    Ki67 unweighted global score (%):

    Ki67 weighted global score (%):

    Number of nuclei counted:

Cold Ischemic and fixation times meet the requirements specified in the latest version of ASCO/CAP guidelines for breast biomarkers.

Methods:

    Primary Antibody:

Ki67 Disclaimer: This assay is not specifically validated for prognostic or predictive purposes in breast cancer [17].
